# Detection of Frozen–Thawed Duck Fatty Liver by MALDI-TOF Mass Spectrometry: A Chemometrics Study

**DOI:** 10.3390/molecules26123508

**Published:** 2021-06-09

**Authors:** Laurent Aubry, Thierry Sayd, Claude Ferreira, Christophe Chambon, Annie Vénien, Sylvie Blinet, Marie Bourin, Angélique Travel, Maeva Halgrain, Véronique Santé-Lhoutellier, Laetitia Théron

**Affiliations:** 1 Institut National de Recherche pour l’Agriculture, l’Alimentation et l’Environnement (INRAE), UR370 Qualité des Produits Animaux, F-63122 Saint Genès-Champanelle, France; laurent.aubry@inrae.fr (L.A.); thierry.sayd@inrae.fr (T.S.); claude.ferreira@inrae.fr (C.F.); christophe.chambon@inrae.fr (C.C.); annie.venien@inrae.fr (A.V.); sylvie.blinet@inrae.fr (S.B.); veronique.sante-lhoutellier@inrae.fr (V.S.-L.); 2 Institut National de Recherche pour l’Agriculture, l’Alimentation et l’Environnement (INRAE), Plateforme d’Exploration du Métabolisme Composante Protéomique (PFEMcp), F-63122 Saint Genès-Champanelle, France; 3 ITAVI, Domaine de l’Orfrasière, F-37380 Nouzilly, France; bourin@itavi.asso.fr (M.B.); travel@itavi.asso.fr (A.T.); maeva.halgrain@inrae.fr (M.H.)

**Keywords:** frozen–thawing cycles, fatty liver, MALDI-TOF mass spectrometry, chemometrics, fraud detection

## Abstract

The marketing of poultry livers is only authorized as fresh, frozen, or deep-frozen. The higher consumer demand for these products for a short period of time may lead to the marketing of frozen–thawed poultry livers: this constitutes fraud. The aim of this study was to design a method for distinguishing frozen–thawed livers from fresh livers. For this, the spectral fingerprint of liver proteins was acquired using Matrix-Assisted Laser Dissociation Ionization-Time-Of-Flight mass spectrometry. The spectra were analyzed using the chemometrics approach. First, principal component analysis studied the expected variability of commercial conditions before and after freezing–thawing. Then, the discriminant power of spectral fingerprint of liver proteins was assessed using supervised model generation. The combined approach of mass spectrometry and chemometrics successfully described the evolution of protein profile during storage time, before and after freezing-thawing, and successfully discriminated the fresh and frozen–thawed livers. These results are promising in terms of fraud detection, providing an opportunity for implementation of a reference method for agencies to fight fraud.

## 1. Introduction

Poultry livers can only be marketed fresh, frozen, or deep-frozen. Therefore, the marketing of defrosted poultry livers is not authorized in France, as stipulated by EU regulation n° 1308/2013 [1]. However, there is a strong irregularity of the market in terms of demands, which is especially true for fatty liver. Indeed, French “foie gras” is a traditional product, a coveted dish that is mostly consumed during the end-of-year celebrations. This high demand in a short period of time could lead to the freezing of livers that are produced during the year, which will be further marketed in a thawed state. This constitutes fraud, and it is a challenge for professionals to tackle, in order to be able to detect this thawed “foie gras”.

Freezing and thawing is known to increase protein oxidation in chicken meat [2] by damaging the cellular ultrastructure, leading to the release of mitochondrial and lysosomal enzymes, haem iron, and other pro-oxidants [3]. Moreover, the storage of duck fatty liver was linked to proteolysis through the identification of proteolytic enzyme by mass spectrometry at different storage times, starting from the first hours postmortem [4], which may be enhanced in the case of freezing because of the formation of ice crystals [5]. Both biological processes, i.e., protein oxidation and proteolysis, can be studied using mass spectrometry. In particular, the protein fingerprint by Matrix-Assisted Laser Dissociation Ionization-Time of Flight mass spectrometry (MALDI-TOF MS) is a method of choice. MALDI-TOF MS is based on the soft ionization of molecules and the measurement of their time of flight, which is proportional to their mass. As such, it can be used to study proteins and the peptides that result from their hydrolysis, and their chemical adducts by the detection of mass shift.

In recent years, MALDI-TOF protein mass spectrometry has become the gold standard method for identifying bacteria in hospitals [6]. The spectrum of proteins that were extracted from a bacterium, named the spectral fingerprint, is compared to a database and a statistical score is given by the system to validate the identification or not. In food science, studies have shown its relevance for the control of the storage time of trout by creating a prediction tool [7]. The discrimination of storage time (0, 3, 7, 9, and 11 days) was achieved with a classification score above 90%. In addition, this spectral method was used to create a database of 54 fish species for adulteration monitoring in the fish industry [8]. Furthermore, the discrimination of defrosted materials in milk that was used in the production of mozzarella di Buffala Campana was performed using MALDI-TOF MS [9]. The proteins that were identified using this approach were defined as a biomarker of milk freshness. As such, they constituted a potential fraud detection, since this product has a Protected Designation of Origin label legislating this aspect of production. More recently, the protein fingerprint successfully predicted the occurrence of normal and PSE-like defect in pork muscle based on a spectral fingerprint of plasma protein samples [10].

In this context, the suitability of MALDI-TOF mass spectrometry to detect thawed fatty liver was studied using the chemometrics approach. The development of a detection tool required several steps. First, fatty liver was sampled to cover the expected variability under commercial conditions: different processing plants origin and a different liver weight range. They were collected in order to optimize the variability of the discriminative model: the effect of a freeze–thaw cycle and storage time. Subsequently, the spectral fingerprint of the fatty liver protein was acquired by MALDI-TOF mass spectrometry, and the resulting spectra were analyzed using chemometrics. The suitability of this combined approach to detect frozen–thawed fatty liver as well as the complementarity of algorithms is further discussed.

## 2. Results and Discussion

### 2.1. Study of the Variability under Commercial Conditions

**Figure 1:**
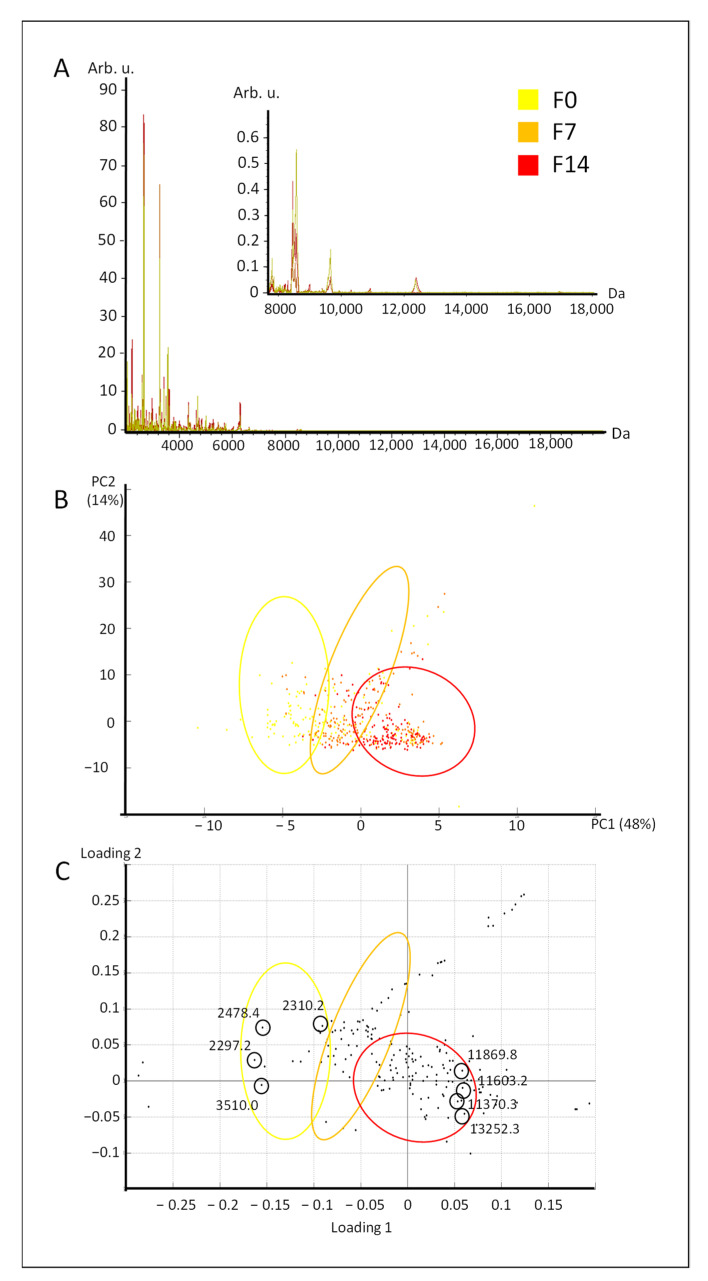
Spectral analysis of fresh livers stored for 0, seven, and 14 days. Mean protein spectra (**A**) from fresh livers stored 0 (F0, in yellow), 7 (F7, in orange), and 14 days (F14, in red). B. Score plot (**B**) and mass loading plot (**C**) of the PCA of protein spectra from fresh livers stored for 0, seven, and 14 days.

The variability under commercial conditions was studied by combining the different origins of processing plants and the different liver weight ranges. The protein spectra from fresh livers that were stored for 0, seven, and 14 days before (F0, F7, and F14) showed a higher number of peaks in the mass range from 2000 to 8000 Da (Figure 1A) than in the mass range from 8000 to 18,000 Da. This is consistent with a previous study on rat and human liver tissue studied by MALDI-TOF imaging [11].

The protein spectra from fresh livers were analyzed using principal component analysis (PCA) (Figure 1B,C). The spatial projection of the protein fingerprint showed a progressive profile from day 0 (F0, in yellow), to day seven (F7, in orange), to day 14 of storage (F14, in red) (Figure 1B). The first two principal components (PC1 and PC2) supported 48% and 14% of variance, respectively. The first principal component mainly explained the spatial distinction between the three kinetic points of storage. The loading plot (Figure 1C) indicated the peaks explaining this projection. The spectral fingerprint of fresh livers that were sampled at day 0 were associated with low masses, which ranged from 2 to 3500 Da approximately. On the opposite side of the PC1 axis, the spectral fingerprint of fresh livers that were stored for 14 days were associated with higher masses, ranging from approximately 11 to 13,000 Da.

A progressive profile of protein fingerprint was observed during fresh liver storage. This result revealed a change in proteins and peptides during this period of time, as previously described in rat liver tissue [11] and trout muscle [7]. In particular, those changes were attributed to proteolysis. This might explain the specific distribution of loadings onto the PC1 axis. Indeed, higher masses were detected with higher intensities at the kinetic point of 14 days of storage, which was the maximum duration in this study (Supplementary data Table S1). Those peaks in the mass range over 11,000 Da might result from a more intense proteolysis of proteins with higher masses; this increased during the storage time of fresh livers. Beyond the mechanisms that those observations might rely on, the combination of MALDI-TOF mass spectrometry and chemometrics successfully revealed a progressive profile of protein spectra during the storage of fresh livers. More importantly, this result was achieved while taking the liver weight range and the processing plant origin into account, since these parameters were equilibrated and randomized.

**Figure 2:**
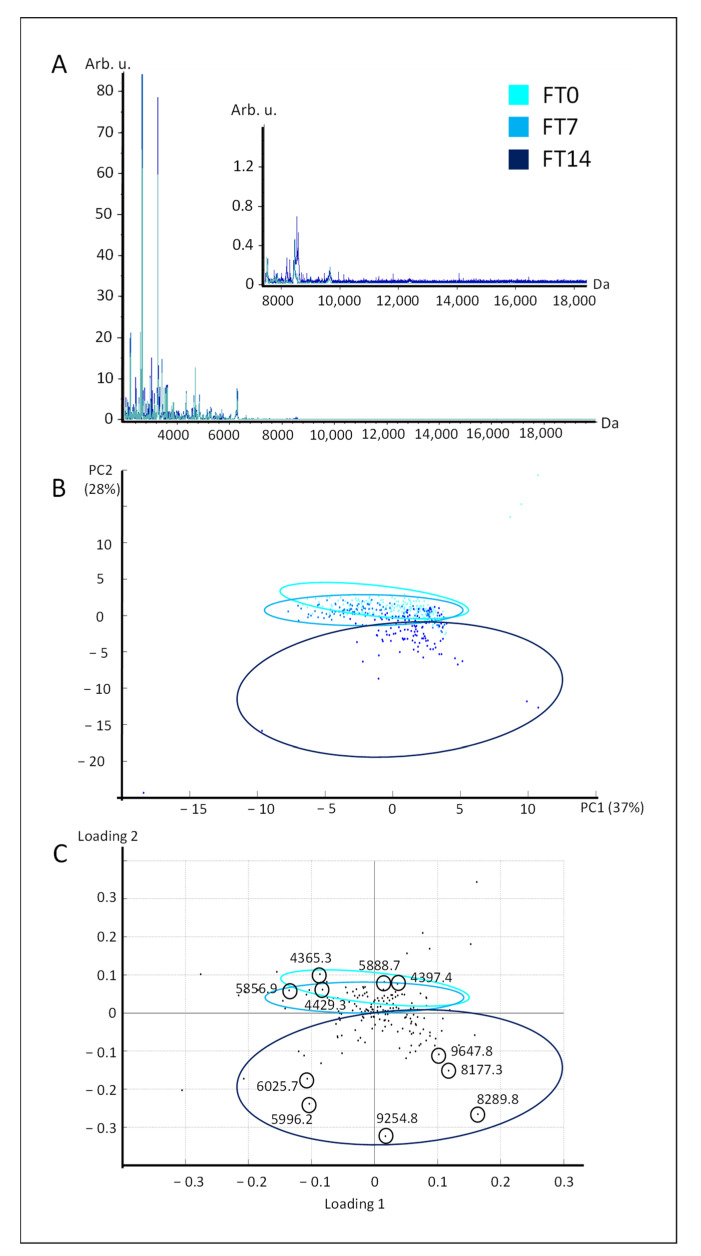
Spectral analysis of frozen–thawed livers stored for 0, seven, and 14 days. Mean protein spectra (**A**) from frozen–thawed livers stored 0 (FT0, in light blue), 7 (FT7, in blue), and 14 days (FT14, in dark blue). B. Score plot (**B**) and loading plot (**C**) of the PCA of protein spectra from frozen–thawed livers stored for 0, seven, and 14 days.

The protein spectra from frozen–thawed livers stored for 0, seven, and 14 days before (FT0, FT7, and FT14) showed a global similar pattern (Figure 2A), which was consistent with what was observed in fresh liver protein spectra (Figure 1A) and rat and human livers [11]. They were analyzed using principal component analysis (PCA) (Figure 2B), and the first two principal components (PC1 and PC2) supported 37% and 28% of variance respectively. The spatial projection of protein spectra from frozen–thawed livers showed a different profile than the one from fresh livers. Indeed, the first two kinetic points of storage, i.e., 0 and seven days (FT0 and FT7), showed a co-projection. The maximum duration of storage, i.e., 14 days (FT14), was spatially separated from the two first kinetic points according to the PC2. The loading plot indicated that the spectral fingerprint of frozen–thawed livers that were sampled at day 0 and 7 were associated with low masses, ranging from approximately 4 to 6000 Da (Figure 2C). On the opposite side of the axis, the spectral fingerprint of frozen–thawed livers that were stored for 14 days were associated with higher masses, ranging from 6 to 9600 Da approximately.

During the storage of frozen–thawed livers, a different protein profile evolution was observed in comparison to fresh livers. The protein fingerprint from day 0 and day 7 (FT0 and FT7) were co-projected, in opposition with the one from day 14 (FT14). In muscle postmortem evolution, the release of enzymes, among which proteases [3], due to structure breakdown, are well described in chicken [12], beef [13], or pork [14] meat, and might explain the present observations. In any case, the study of the expected variability of frozen–thawed fatty livers using the combination of MALDI-TOF mass spectrometry and chemometrics was conclusive. Indeed, a progressive profile was observed, consistent with bibliography, and, as previously discussed, while taking the liver weight range and the processing plant origin into account.

Describing and covering the expected variability under commercial conditions is essential in developing a fraud detection method. Indeed, the statistical information covered by the studied criterion, i.e., between fresh and frozen–thawed livers, has to be higher than the one within each class. This is called the “data-expansion strategy”, and it helps to avoid over-fitting [15], which is mandatory for the detection method to be reliable and usable by the fraud control laboratories.

### 2.2. Discrimination of Fresh and Frozen–Thawed Livers

Once the expected variability under commercial conditions was determined, the liver protein fingerprints at different storage times were combined to evaluate the discrimination of fresh and frozen–thawed livers. To do that, the protein spectra from different processing plants, the three liver weight ranges, and the three storage kinetic point were combined to create two groups: fresh livers, named “F”, and frozen–thawed livers, named “FT”. These two spectral classes were further analyzed using three different algorithms: the Quick Classifier (QC), Supervised Neural Network (SNN), and Genetic Algorithm (GA). The results were expressed as cross-validation and recognition capability percentage (Table 1).

The three approaches showed similar results in terms of cross-validation, i.e., internal validation. The scores ranged from 69.7% to 93.2% when cross-validation was calculated per classes, and from 77.6% to 86.4% when it was calculated globally, for the two classes. On the contrary, the recognition capability was quite different between the three algorithms. The SNN resulted in a 49.4% of recognition capability, which can be considered as a low score, since the algorithm is expected to badly classify more than half of the spectra. On the other hand, QC and GA resulted in a much higher score of 88.2% and 93.3%, respectively.

The different performance showed by the three algorithms to discriminate fresh from frozen–thawed livers can be explained by the different computing principles that they rely on. Indeed, the QC algorithm discriminates classes using the statistical differences between the peaks. The selection of peaks included in the calculation is carried out according to their *p*-values after an automatic detection of peak number by the algorithm. Subsequently, the SNN algorithm is an iterative method that is based on the characteristics of data distribution [16]. Prototypes are defined based on peaks that can describe the best of each class. Finally, the GA is a random algorithm that is meant to mimic the natural evolution [17], which considers each spectrum as a chromosome and each peak as a gene. The overall principle is to recover the family belonging, which means the class discrimination, based on the idea of the evolution of the fittest individual [18]. All of these differences are the reason why these algorithms are complementary. When it comes to developing a detection method, it is better to compare different approaches to determine which one is the more suitable. Based on these results, i.e., the cross-validation and recognition capability percentages, the QC and GA approaches are the most efficient algorithms for successfully discriminating fresh from frozen–thawed livers.

The number of peaks involved in the calculation to discriminate fresh and frozen–thawed livers is another criterion to take into account. The QC, SNN, and GA results are based on the intensities of 21, one, and five peaks, respectively (Table 2). The SNN algorithm’s results confirmed its irrelevancy in this study, since it did not show a predictive value in the present case. Indeed, the results are based on only one peak intensity, which renders the approach difficult to rely on, since it may be more prone to technical variability and/or environmental contamination for instance. Following the same principle, the QC algorithm may not be more suitable for discriminating fresh from frozen–thawed livers, since its results are based on 21 peaks intensities. From a practical point of view, it means that all of these 21 peaks will have to be detected for the method to be successful. The weight criteria, which indicate the coefficient of each peak involvement in the calculation, ranged from approximately one to almost 10. It is expected that the QC model may lose most of its power if a peak with a higher weight was not detected, and so not included in the calculation. Finally, the GA algorithm is based on five peak intensities showing similar weight, from 0.3 to 1. Furthermore, the peaks are part of the best defined mass range, being lower than 10,000 Da, which make them more likely to be detected with high reproducibility. In conclusion, from these results, it can be deduced that the GA algorithm would be a successful approach for discriminating fresh from frozen–thawed livers based on the protein fingerprint.

The involvement of three peaks in the calculation of two algorithms is additional information given by the comparative list of peaks (Table 2). Indeed, the masses 4262.37 Da, 4743.13 Da, and 6640.20 Da are part of the results that are given by the QC and GA algorithms. This observation might emphasize the importance of these peaks in the discrimination of fresh and frozen–thawed livers since they were defined as potential markers using different approaches. The area under ROC (Receiver Operating Characteristic) curve (AUC) is commonly used to evaluate the accuracy of discrimination of a single criterion [19], and to increase it when used in combination with other approaches [20], as is the case in the present study. The AUC of the three given peaks of interest, i.e., 4262.37 Da, 4743.13 Da, and 6640.20 Da, were found to be 0.76, 0.88, and 0.82, which confirmed their high accuracy in discriminating fresh and frozen–thawed livers.

This result is of great importance since it opens up new possibilities of implementation of the detection method in fraud control laboratories. Indeed, the identification of these peaks might enable the development of specific tests, such as, for instance, an antigenic test. Even if most of the fraud control laboratories are now equipped with MALDI-TOF mass spectrometer, especially because of the bacteria identification field, it may be interesting for them to have more control possibilities.

## 3. Materials and Methods

### 3.1. Chemical and Reagents

The α-cyano-4-hydroxy-cinnamic acid matrix (CHCA, 99%), trifluoroacetic acid (TFA, 99%), acetonitrile (≥99.9%), and methanol HPLC grade (≥99.9%) were purchased from Sigma-Aldrich (Sigma-Aldrich, Steinheim, Germany). The protein calibration standard II was purchased from Bruker Daltoniks (Bruker Daltoniks, Bremen, Germany). Purification column Spin Tubes (C18 Peptide Cleanup) were obtained from Agilent Technologies (Agilent Technologies, Wilmington, DE, USA).

### 3.2. Experimental Design and Liver Sampling

The study was based on 228 duck fatty livers (Table 3). The sampling was randomly done in three processing plants on the commercial lines to be representative of the marketed products. The livers were weighted and distributed into three classes: less than 500 g, between 500 and 600 g, and more than 600 g. They were sampled before freezing, namely “Fresh”, right after chilling (F0), after seven or 14 days of storage at 4 °C under vacuum (F7 and F14), and after six-months freezing and thawing, namely “Frozen–thawed”, right after thawing (FT0), after seven or 14 days of storage at 4 °C (FT7 and FT14).

### 3.3. Proteins Extraction

Liver proteins were extracted according to Sayd et al. [21] and pre-purified using Spin Tubes (C18 Agilent Peptide Cleanup, Agilent Technologies, Wilmington, DE, USA), according to the manufacturer’s instructions. Briefly, 200 μL of acetonitrile was used to wet the resin phase, and the tubes were centrifuged at 1500× *g* for 1 min.; this step was then repeated. Next, the equilibration of resin was performed using 200 μL of 0.5% trifluoroacetic acid in 5% acetonitrile, and then tubes were centrifuged at 1500× *g* for 1 min.; this step was then repeated. Subsequently, the protein samples were loaded on the resin, and the resin was washed using 200 μL of 0.5% of trifluoroacetic acid in 5% acetonitrile; this step was repeated twice. The proteins were eluted using 20 μL of 70% acetonitrile with 0.1% trifluoroacetic acid, dried, and resuspended in 20 μL 0.2% trifluoroacetic acid for the MALDI-TOF mass spectrometry analysis.

### 3.4. MALDI-TOF Mass Spectrometry Protein Fingerprinting

The liver protein fingerprints were acquired according to [10]. For MALDI-TOF acquisition, 1 μL of pre-purified plasma proteins was manually spotted in triplicate on a polished steel target (MTP 384 Target Plate Polished Steel, Bruker Daltonics GmbH, Bremen, Germany). The matrix used was α-cyano-4-hydroxy-cinnamic acid (CHCA) matrix at 7 mg/mL in water/acetonitrile 50:50 (*v*/*v*) with 0.2% trifluoroacetic acid, at a ratio of 1:1 with the sample. The protein fingerprinting was acquired on an Autoflex Speed MALDI-TOF/TOF mass spectrometer with a Smartbeam laser, utilizing FlexControl (version 3.4) software (Bruker Daltonics GmbH, Bremen, Germany). A total of 3500 spectra were randomly accumulated per sample, and each acquisition was done in triplicate. The laser power was constant for all of the samples to avoid any bias in the spectra comparison, and the laser focus was set at medium. The linear mode was set to acquire protein spectra, at a mass range of *m*/*z* 1000–20,000, with a sampling rate of 0.31 GS/s. The acquisition deflection was set at *m*/*z* 1000. The detector gain was set at 2500 V, the ion source voltage 1 at 19.56 kV, the ion source voltage 2 at 18.11 kV, and the lens voltage at 7 kV. An external calibration of spectra was done through the deposition of a protein standard (Protein Calibration Standard I, Bruker Daltonics, Bremen, Germany) before each measurement on the same target.

MALDI-TOF spectra were processed using FlexAnalysis (version 3.4) software (Bruker Daltonics GmbH, Bremen, Germany). The baseline was subtracted using the TopHat algorithm, with a 5% minimal baseline width. Spectra were smoothed using Savitzky-Golay algorithm with a 1 Da width and five cycles. The spectra were re-calibrated with a maximum peak shift of 0.1% and a minimum matching score of 30% of the most represented peaks, in order to take the differences between classes into account. Individual spectra were normalized using the TIC value. Peak picking was applied using the peak intensities on individual spectra, with a signal to noise ratio of 3.

### 3.5. Chemometrics

Chemometrics, i.e., principal component analysis and classification model generation, were performed using the ClinProTools^TM^ software (version 3.0, Bruker Daltonics, Bremen, Germany). The samples were analyzed in triplicate after randomized protein extraction and spectra acquisition.

Principal Component Analysis (PCA) was calculated to visualize sample projection on the score plot and the variables explaining the projection of the loading plot.

Classification model generation was performed with three complementary algorithms: the Quick Classifier (QC), the Supervised Neural Network (SNN) [16], and the Genetic algorithm [17]. The algorithm automatically determined the number of peaks that were involved in the calculation of SNN and the number of prototype detection. The evaluation results of the models were expressed as “Cross-validation” and “Recognition capability”. Cross validation indicates the model’s reliability and its future performance. It is calculated by randomly dividing the spectral data into two datasets: the one used to generate the model and one used to validate it. This operation is iterated multiple times to obtain the cross validation value. Recognition capability measures the model’s ability to correctly classify the spectral data that are used to generate the model. The calculation consists of testing each spectrum using the model itself and dividing the number of correct spectra by the total number of spectra, giving a percentage of correctly classified spectral data. Internal cross-validation was calculated using 20% of the spectral data, randomly selected, and then performed on ten iterations. The analysis of the operational characteristic of the receiver (ROC) was performed for each relevant peak, which is involved in at least one machine learning result. The resulting area under the ROC curve (AUC) was used to assess the peak relevance: from 0 (non-discriminant peak) to 1 (highly discriminant peak).

## 4. Conclusions

The protein fingerprint using MALDI-TOF mass spectrometry combined with chemometrics has fulfilled both of the objectives of this study. First, the expected variability under commercial conditions was successfully described during the storage of fresh and frozen–thawed fatty livers. These results provided new possibilities of further research by highlighting the importance of storage conditions and their consequences on protein spectral fingerprint. Subsequently, a method to discriminate fresh and frozen–thawed fatty livers was successfully designed, while taking the commercial variability into account. This also opens up new possibilities through the further identification of the peaks involved that could help in defining complementary control fraud tools.

## Figures and Tables

**Table 1:** The evaluation results of the QC, SNN, and GA algorithms to discriminate fresh and frozen–thawed livers based on the protein fingerprint.

		QC	SNN	GA
Cross-validation	Fresh livers	83.1%	69.7%	79.6%
	Frozen–thawed livers	84.1%	85.4%	93.2%
	Global	83.6%	77.6%	86.4%
Recognition capability		88.2%	49.4%	93.3%
Number of peaks involved		21	1	5

**Table 2:** A list of peaks involved in the calculation of the discrimination of fresh and frozen–thawed livers.

				Weight			AUC
Mass, in Da	Fresh Livers	Frozen–Thawed Livers	*p*-Value	QC	SNN	GA	
2073.37	7.96 ± 1.38	5.99 ± 0.91	<0.000001			0.32	0.65
2202.14	15.62 ± 4.75	24.04 ± 4.72	<0.000001	5.3			0.96
2596.51	20.00 ± 3.37	18.28 ± 3.69	<0.000001			0.24	0.61
2691.06	6.77 ± 0.44	9.88 ± 0.97	<0.000001	9.4			0.74
2965.47	5.34 ± 0.56	4.29 ± 0.39	<0.000001	5.3			0.79
3541.38	3.75 ± 0.31	3.91 ± 0.22	0	1.4			0.78
3555.07	18.97 ± 5.70	11.84 ± 3.62	<0.000001	5.9			0.77
3570.96	7.02 ± 0.96	5.89 ± 1.12	<0.000001	1.09			0.76
3606.55	10.06 ± 1.52	8.57 ± 1.17	<0.000001	3.6			0.82
4262.37	3.48 ± 0.23	3.97 ± 0.35	<0.000001	1.8		0.69	0.76
4316.21	9.4 ± 0.98	9.5 ± 1.21	0.0000378	4.5			0.79
4347.77	4.21 ± 0.36	3.8 ± 0.33	<0.000001	1.6			0.70
4414.55	2.55 ± 0.32	3.17 ± 0.63	<0.000001		0.06		0.63
4628.01	5.13 ± 0.85	7.07 ± 0.91	<0.000001	2.2			0.82
4743.13	3.24 ± 0.64	3.89 ± 0.54	<0.000001	7.2		1.06	0.88
4822.69	3.29 ± 0.65	7.20 ± 1.65	<0.000001	1.7			0.84
4833.79	1.99 ± 0.23	1.78 ± 0.19	<0.000001	2.7			0.86
4990.13	4.13 ± 1.03	2.33 ± 0.31	<0.000001	1.7			0.83
5160.81	2.19 ± 0.21	2.49 ± 2.31	<0.000001	1.4			0.77
5225.46	2.37 ± 0.17	2.65 ± 0.25	<0.000001	1.6			0.74
5460.16	2.3 ± 0.28	2.47 ± 0.31	0.00000193	3.5			0.72
6243.72	0.96 ± 0.10	0.99 ± 0.10	<0.000001	2.2			0.82
6283.88	5.84 ± 1.14	8.12 ± 1.27	<0.000001	1.7			0.82
6640.20	1.43 ± 0.16	1.41 ± 0.18	<0.000001	9.4		0.86	0.82

Peak intensities, in arbitrary unit, in F and FT groups are expressed as mean ± standard error of three measurements.

**Table 3:** The experimental design of the study.

	Storage Time (in Days)	Weight Range (in Grams)	Number of Livers
Fresh	F0	<500	15
		500–600	15
		>600	15
	F7	<500	10
		500–600	10
		>600	10
	F14	<500	15
		500–600	15
		>600	15
Frozen–thawed	FT0	<500	12
		500–600	12
		>600	12
	FT7	<500	12
		500–600	12
		>600	12
	FT14	<500	12
		500–600	12
		>600	12

## Data Availability

Data is contained within the article or supplementary material.

## Supplementary Materials

Table S1: List of peaks detected in fresh and frozen-thawed livers.

## Sample Availability

Samples are available from the authors.
